# Mechanisms of Mitochondrial Apoptosis-Mediated Meat Tenderization Based on Quantitative Phosphoproteomic Analysis

**DOI:** 10.3390/foods11233751

**Published:** 2022-11-22

**Authors:** Jiaying Zhang, Shuangshuang Wang, Wupeng Ge

**Affiliations:** College of Food Science and Engineering, Northwest A&F University, Yangling 712100, China

**Keywords:** quantitative phosphoproteomic, proteins, apoptosis, tenderness, postmortem, muscle

## Abstract

This study investigates the mechanism of phosphorylation in the regulation of apoptosis-mediated meat tenderization during postmortem aging. The results found that the pork muscle exhibited apoptotic potential at early postmortem (48 h) and showed more tenderness at late postmortem, as evidenced by the increase in mitochondrial membrane permeability (MMP), Ca^2+^ level, reactive oxygen species (ROS) content, and caspases activity at 0 h to 48 h, and decreases in ATP level at 0 h to 24 h and shear force at 12 h to 120 h (*p* < 0.05). Phosphoproteomic analysis revealed that phosphorylation regulated apoptosis by modulating ATP and calcium bindings as well as apoptotic signaling, which occurred within early 12 h and mainly occurred at 12 h to 48 h postmortem. Moreover, differential expression of phosphoproteins demonstrated that phosphorylation regulated oxidative stress-induced apoptosis and rigor mortis, thereby promoting the development of meat tenderness. Our results provide insights into the roles of phosphorylation in various physiological processes that affect meat tenderness.

## 1. Introduction

Meat quality is of great importance in the meat industry, which has to satisfy the expectations of consumers and, for them, meat tenderness is, and will always be, the primary quality attribute [1]. It is well documented that ultimate tenderness depends on the extent of proteolysis of key target proteins within muscle fibers [2]. Caspases are a family of cysteine aspartate-specific proteases that play crucial roles in apoptosis [3]. Apoptosis occurs immediately after animal bleeding, which is confirmed to depend on the mitochondrial pathway [4]. Several studies have reported the contributions of mitochondrial apoptosis to meat tenderization [5,6,7,8], and have suggested that the pH was one of the influencing factors of apoptosis [9]. Nevertheless, the underlying mechanism of mitochondrial apoptosis-mediated meat tenderization is still not completely understood.

Protein phosphorylation is the most common post-translational modification, and it plays regulatory roles in the decline of muscle pH, the proteolysis of myofibrillar proteins and the development of meat tenderness during postmortem aging [10]. Recent studies have reported that myofibrillar protein phosphorylation is negatively associated with meat tenderness [11,12]. D’Alessandro and Zolla [13] demonstrated that phosphorylation regulates meat tenderness possibly by regulating the protective function of heat shock proteins with anti-apoptotic activity. Chen et al. [14] and Huang et al. [15] reported that the phosphorylation status of some proteins could be closely related to meat’s ultimate pH and tenderness. Thus, it is reasonable to speculate that phosphorylation-regulated meat tenderization is related to apoptosis in postmortem muscles. Nevertheless, little is known on the relationship between apoptosis and protein phosphorylation. Given that protein phosphorylation and dephosphorylation are involved in the regulation of a variety of important cellular physiological processes, such as metabolism, signal transduction and apoptosis [16], it could be postulated that phosphorylation regulates apoptosis-mediated meat tenderization.

In this work, with the aim of elucidating the mechanism of phosphorylation in the regulation of apoptosis-mediated meat tenderization, a quantitative MS-based phosphoproteomic analysis of postmortem muscles was performed. The results of this study enhance our understanding of the roles of phosphorylation in apoptosis-mediated meat tenderization, which can be helpful for meat quality control in the future.

## 2. Materials and Methods

### 2.1. Meat Samples

Six pigs (Berkshire × Bamei × Neijiang) with average weights of 120 kg fed on the same diet in the same batch were slaughtered by trained personnel at a pig breeding farm on the campus of Northwest A&F University (Yangling, China). All animal care procedures and experimental methods adhered to the guidelines set forth by the Institutional Animal Care and Use Committee of Northwest A&F University (2015-1-138). After bleeding, approximately 200 g of pork *longissimus dorsi* (LD) muscle samples were excised from the carcasses at 0, 6, 12, 24, 48, 120 and 168 h at 4 °C, respectively [17]. After removing visible fat and connective tissue, approximately 120 g of LD samples were applied for mitochondria isolation and shear force detection. Moreover, the remainder of LD samples were frozen in liquid nitrogen and then stored at −80 °C until analysis.

### 2.2. Apoptosis Related Factors and Tenderness 

#### 2.2.1. Mitochondrial Membrane Permeability (MMP) and Ca^2+^ Level

The isolation of mitochondria was performed strictly according to a previous method [17]. MMP and Ca^2+^ level was detected according to Zhang et al. [17] and Wang et al. [18], respectively. Briefly, the mitochondrial protein concentration was diluted to 0.5 mg/mL. The light absorption value was determined at 540 nm by UV spectrophotometry (UV2550, Shimadzu, Kyoto, Japan). Mitochondrial Ca^2+^ level was detected by using a flame atomic spectrophotometer. Briefly, mitochondrial proteins (2 mg) were mixed with ultrapure water and concentrated nitric acid at the ratio of 1:1:2 (*v/v/v*). The mixture was digested for 1 h. The lanthanum oxide solution (20 g/L) was then added to the digested sample. The final volume of the digested sample was adjusted to 10 mL with 1% nitric acid. The absorbance was measured using a flame atomic absorption spectrophotometer (iCE 3000, Thermo Scientific, Waltham, MA, USA).

#### 2.2.2. ATP Level

The mitochondrial ATP level was determined according to a previous study [6] by using an ATP Assay Kit (A095-1-1, Nanjing Jiancheng Bioengineering Institute, Nanjing, China). In brief, mitochondrial proteins (30 μL) were incubated with substrate I (100 μL), substrate II (200 μL) and accelerator (30 μL) for 30 min at 37 °C. Subsequently, precipitator (50 μL) was added and the samples were centrifuged at 3500× *g* for 5 min. Then, the supernatant (300 μL) was mixed with a color-developing agent (500 μL). Finally, the terminating agent (500 μL) was added. The absorbance was detected at 636 nm by UV spectrophotometry. 

#### 2.2.3. Reactive Oxygen Species (ROS) Content

ROS content was detected according to a previous study [7]. In brief, 1 g of LD muscle was homogenized in 6 mL of buffer solution I (10 mM Tris-HCl, 10 mM sucrose, 0.1 mM EDTA-2Na, 0.8% (*w/v*) NaCl, pH 7.4) and then centrifuged at 3000× *g* for 15 min. The supernatant (100 μL) was mixed with 100 μL of buffer solution II (buffer solution I + 10 μM DCFH-DA) and then incubated for 30 min. The fluorescence value was determined at 450 nm with a M200 Multiskan Spectrum Instrument (M200, TECAN, Switzerland). The ROS content was calculated according to the following formula, in which V1: fluorescence value before incubation; V2: fluorescence value after incubation; T: incubation time; and C: proteins concentration.

$$\mathrm{ROS}\;\mathrm{content}=\frac{V1-\;V2}{T}/C$$

#### 2.2.4. Caspases Activity

The Activity Colorimetric Assay Kit (K105-100, BioVision, San Francisco, CA, USA) was used to determine the caspases activity according to the manufacture’s protocol, strictly. Briefly, 200 mg of LD muscle was homogenized in 0.5 mL lyses buffer and then centrifuged at 15,000× *g* for 30 min. The supernatant was added to protease assay buffer containing caspase-9 and caspase-3 substrates in 96-well plates, which were then incubated for 2 h at 37 °C. The caspases activity was determined at 405 nm on the microplate reader (Multiskan Spectrum, Thermo Scientific, USA).

#### 2.2.5. Shear Force

This assay was measured as previously described [7]. About 70 g meat samples were packed in individual bags and immersed in 80 °C water until the entire sample reached an internal temperature of 75 °C. Subsequently, the meat samples were cooled to room temperature and three cores (1.27 cm in diameter) were cut from the steaks, parallel to the orientation of the muscle fibers. Samples were measured using a tenderometer (C-LM3B, Harbin Electrical Measuring Instrument Co., Ltd., Harbin, China). Each sample was performed at least thrice.

### 2.3. Protein Extraction and Trypsin Digestion

Protein extraction was performed as reported previously [19]. In brief, 1 g of frozen muscle sample was grinded into powder in liquid nitrogen. Subsequently, a 4-times volume of lysis buffer (8 M urea, 1% protease inhibitor, 1% phosphatase inhibitor, 50 μM PR-619, 3 μM trichostatin A, 50 mM nicotinamide) was added, and the samples were sonicated three times. The lysates were centrifuged at 12,000× *g* for 10 min at 4 °C. The supernatants were collected, and the protein concentration was determined using the BCA Protein Assay kit (23225, Pierce, ThermoFisher, Waltham, MA, USA). The protein samples were diluted with 100 mM NH_4_HCO_3_ so that the final concentration of urea was lower than 2 M. The samples were reduced with 5 mM dithiothreitol for 30 min at 56 °C and alkylated with 11 mM iodoacetamide for 50 min at 25 °C in the dark. Subsequently, the proteins were digested and incubated with trypsin (V5111, Promega, Madison, WI, USA) at a ratio of 1:50 (trypsin/protein, m/m) overnight at 25 °C.

### 2.4. Tandem Mass Tags (TMT) Labeling

After trypsin digestion, the peptides were desalted with the Strata × C18 column (8B-S001-1SN, Phenomenex, Torrance, CA, USA) and vacuum freeze-dried. The TMT kit (90064, ThermoFisher, USA) was used to label proteins according to the manufacturer’s protocol. In brief, 1 unit of TMT labelling regent was thawed and dissolved in acetonitrile. The peptides were dissolved in 0.5 M triethylammonium bicarbonate and incubated with TMT labelling regent for 2 h at room temperature. The labeled peptides were desalted and dried by vacuum centrifugation.

### 2.5. High Performance Liquid Chromatography (HPLC) Fractionation

The dried peptides were fractionated by high-pH reverse-phase HPLC coupled with the Agilent 300Extend C18 column (particle size, 5 μm; internal diameter, 10 mm; length, 250 mm). In brief, the peptides were separated into 60 fractions using a gradient of 8% to 32% acetonitrile (pH 9.0) for 60 min. The peptides were pooled into four fractions and dried by vacuum centrifugation.

### 2.6. Phosphopeptide Enrichment and LC-MS/MS Analysis

This assay was performed as reported previously with minor modifications [20]. The dried peptides were dissolved in buffer containing 50% acetonitrile/0.5% trifluoroacetic acid. The mixture was loaded on immobilized metal affinity chromatography (IMAC) microspheres and incubated under vibration. After incubation, the IMAC microspheres were washed and the phosphopeptides were eluted with 10% NH_4_OH. The phosphopeptides were dried, desalted and used for LC-MS/MS analysis.

The peptide fractions were separated by the EASY-nLC 1200 ultra-high performance liquid chromatography system (EASY-nLC 1200, ThermoFisher, USA). The mobile phase A consisted of 0.1% formic acid/2% acetonitrile, and the mobile phase B consisted of 0.1% formic acid/90% acetonitrile. The chromatographic gradient increased from 4% to 22% mobile phase B over 38 min, followed by 22% to 32% for 14 min and an increase to 80% in 4 min and a plateau at 80% for 4 min at a constant flow rate of 500 nL/min. After separation, the peptides were injected into an NSI iron source, followed by analysis with Orbitrap Exploris™ 480 software (Exploris™ 480, ThermoFisher, USA). An electrospray voltage of 2.3 kV was applied. An MS scan range of 350 to 1400 m/z was applied with a resolution of 60,000. The peptides were selected for MS/MS analysis by 28% normalization collision energy, and ion fragments were detected with a resolution of 15,000. The automatic gain control was set at 100%, and the signal threshold was set to 1 × 10^4^ ions/s.

### 2.7. Database Search

The MS/MS data were processed with Proteome Discoverer software (v2.4.1.15) and subjected to database searching using the Mascot 2.3 server. The database was Blast_Sus_scrofa_9823_PR_20210806.fasta, which contained 49,792 sequences. Trypsin was set as the cleavage enzyme, which allowed up to two missed cleavages. Carbamidomethyl (C), TMT6plex (peptide N-Terminus) and TMT6plex (K) were designated as the fixed modifications, and oxidation (M) and phospho (S, T, Y) were designated as the variable modifications. The false discovery rate (FDR) was set at less than 1%. The phosphorylation sites with a localization probability higher than 0.75 were accepted. To test the reproducibility of this experiment, the quantification of phosphorylation ratios in each replicate was based on the ratio of the summed intensity of the matched spectra. Pearson correlation coefficient (PCC), principal component analysis (PCA) and average relative standard deviation (RSD) were used to evaluate the reproducibility of the relative protein quantitation. 

### 2.8. Statistical Analysis

The data of MMP, Ca^2+^ level, ATP level, ROS content, caspase-9 and caspase-3 activity, and shear force among different aging periods (0, 6, 12, 24, 48, 120 and 168 h) were analyzed by using the Analysis of Variance (ANOVA) procedure. The Duncan’s multiple range test (*p* < 0.05) was used to compare the differences between the mean values. Each assay was performed at least thrice. Molecular functions, biological processes and cellular components of differentially phosphorylated proteins (*p* < 0.05) were classified by Gene Ontology (GO) annotations using the Uniprot database http://www.uniprot.org/ (accessed on 8 October 2021). The subcellular localizations were analyzed by WolF Psort 3.0 software https://psort.hgc.jp/ (accessed on 8 October 2021). The differentially expressed proteins were analyzed with PTM-Biolab http://www.ptmbiolab.com/#/auth/login (accessed on 8 October 2021). For the protein–protein interaction analysis, the interaction networks of the identified proteins were obtained from the STRING 11.0 database. The interaction network only presented proteins with connections.

## 3. Results

### 3.1. Changes in Mitochondrial Apoptosis Related Factors and Tenderness in Postmortem Muscle

**Figure 1:**
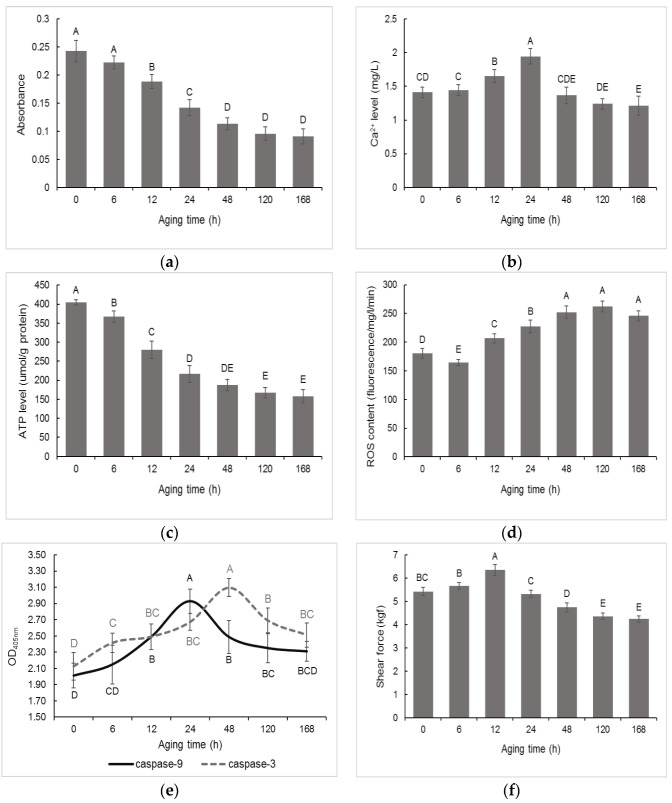
Changes in apoptosis related factors and tenderness in postmortem pork muscle. (**a**) mitochondrial membrane permeability (MMP), (**b**) Ca^2+^ level, (**c**) ATP level, (**d**) reactive oxygen species (ROS) content, (**e**) caspase-9/-3 activity, (**f**) shear force. Different capital letters represent the difference between aging (*p* < 0.05).

To understand the mitochondrial apoptosis and meat tenderness in postmortem pork muscle, MMP, mitochondrial Ca^2+^ level, mitochondrial ATP level, ROS content, caspase-9 and caspase-3 activity, and shear force were investigated first. The MMP assay indicated that the lower absorbance values reflected increased light penetration through the mitochondria, which suggests higher MMP. In Figure 1a, the MMP was increased at 6 h to 48 h (*p* < 0.05) but the increase was insignificant at 48 h to 168 h postmortem. In Figure 1b, mitochondrial Ca^2+^ levels were increased at 6 h to 24 h and decreased at 24 h to 48 h (*p* < 0.05) postmortem. In addition, mitochondrial ATP levels were decreased at 0 h to 24 h (*p* < 0.05) (Figure 1c). ROS content was decreased at 0 h to 6 h and increased at 6 h to 48 h (*p* < 0.05) postmortem (Figure 1d). The caspase-9 activity and caspase-3 activity reached maximum levels at 24 h and 48 h, respectively (Figure 1e). Further, the shear force was increased at 0 h to 12 h but decreased at 12 h to 120 h (*p* < 0.05) postmortem.

### 3.2. Identification of Phosphorylated Differentially Expressed Proteins in Postmortem Muscle

**Figure 2:**
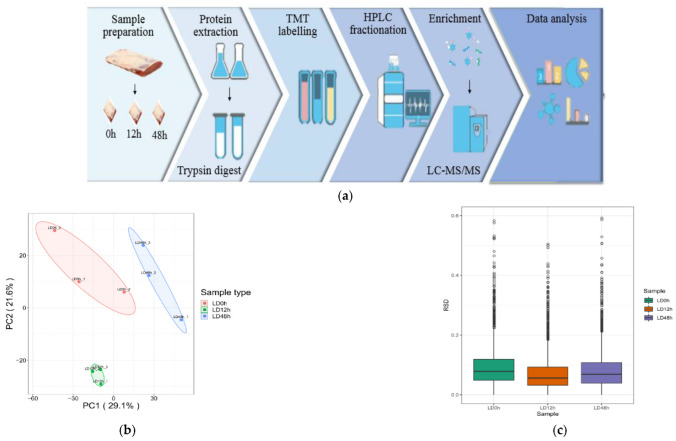

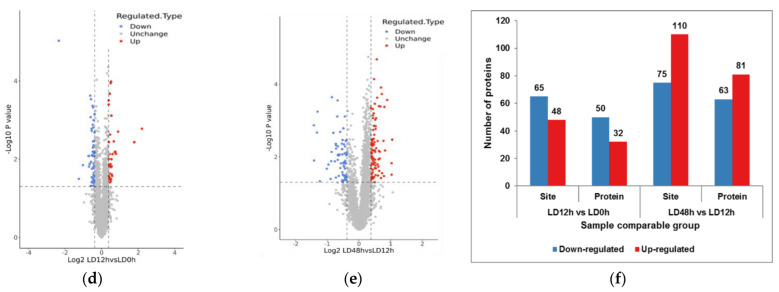
Identification of differentially expressed proteins in postmortem pork muscle. (**a**) The schematic overview for the quantitative analysis of pork muscle proteome and phosphoproteome at 0 h to 48 h postmortem. (**b**) Two-dimensional scatter plot of PCA distribution of protein quantitation. (**c**) RSD of protein quantitation. (**d**) Volcano plot of the differentially expressed proteins between 0 h and 12 h postmortem. (**e**) Volcano plot of the differentially expressed proteins between 12 h and 48 h postmortem. (**f**) Quantitative analysis of differentially expressed proteins in different sample comparable groups.

The present study indicated that the mitochondrial apoptosis-related factors were changed mainly at 0 h to 48 h postmortem. To elucidate the phosphorylation events involved in regulation of apoptosis in postmortem pork muscle, LC-MS/MS-based quantitative phosphoproteomic analysis at 0, 12 and 48 h postmortem was conducted. The schematic overview of this assay is shown in Figure 2a. Figure 2b,c exhibited good quantitative repeatability. Volcano plots of the differentially expressed proteins between 0 h and 12 h, as well as 12 h and 48 h postmortem, are shown in Figure 2d,e, respectively. From Figure 2f, a total of 113 phosphorylation sites with 82 phosphoproteins and 185 phosphorylation sites with 144 phosphoproteins were identified at 0 h to 12 h and 12 h to 48 h postmortem, respectively. Among these, 65 phosphorylation sites of 50 phosphoproteins were down-regulated, while 48 phosphorylation sites of 32 phosphoproteins were up-regulated at 0 h to 12 h postmortem. Furthermore, 75 phosphorylation sites assigned to 63 phosphoproteins and 110 phosphorylation sites assigned to 81 phosphoproteins were down-regulated and up-regulated at 12 h to 48 h postmortem, respectively (Supplemental Table S1). 

### 3.3. Functional Analysis of Differentially Expressed Proteins in Postmortem Muscle

**Figure 3:**
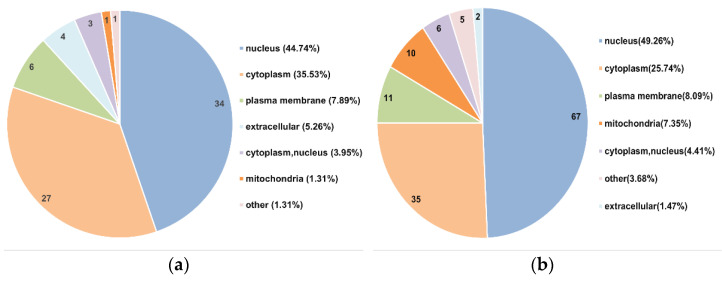

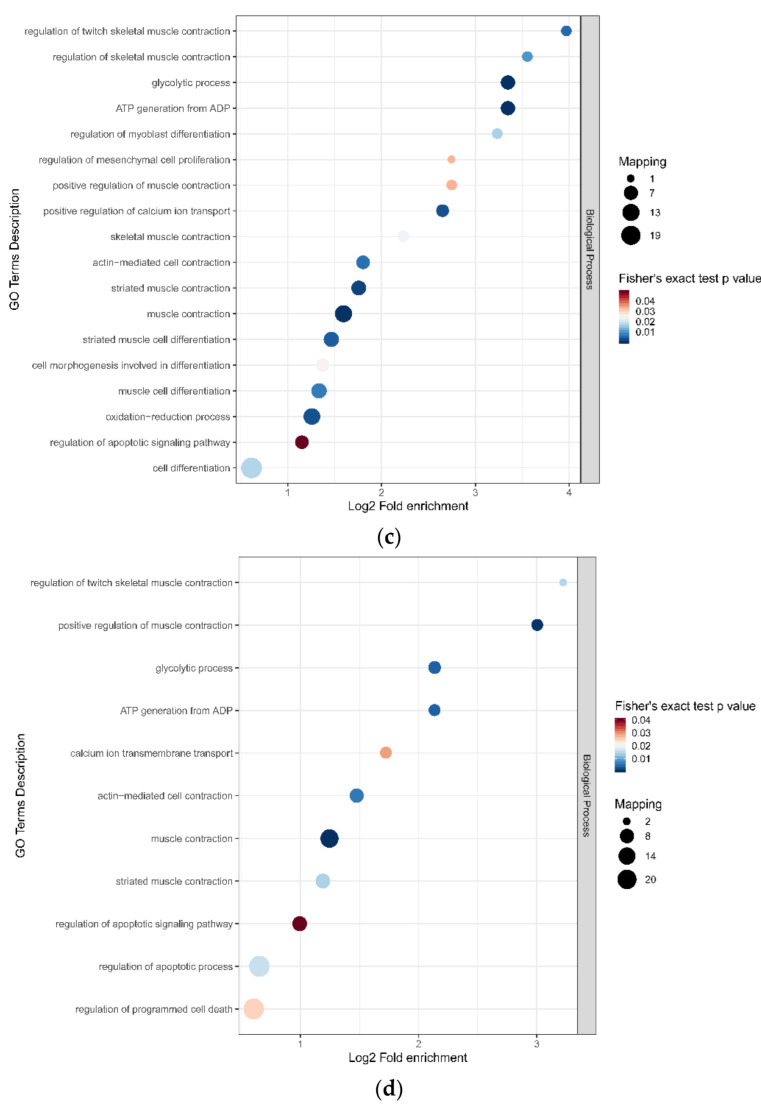
Functional analysis of differentially expressed proteins in postmortem pork muscle. (**a**,**b**) Subcellular localizations of differentially expressed proteins at 0–12 h postmortem and 12–48 h postmortem, respectively. (**c**,**d**) GO functional enrichment bubble plot of the differentially expressed proteins at 0–12 h postmortem and 12–48 h postmortem, respectively.

To thoroughly understand the functions of the differentially expression proteins, their subcellular localizations were analyzed with WolF Psort 3.0 software https://psort.hgc.jp/ (accessed on 8 October 2021). As shown in Figure 3a,b, most differentially expressed proteins were annotated as nucleus (34 at 0–12 h and 67 at 12–48 h), followed by the cytoplasm (27 at 0–12 h and 35 at 12–48 h) and plasma membrane (6 in at 0–12 h and 11 at 12–48 h) in two comparable groups. In addition, 4, 3 and 1 proteins/protein were located in the extracellular, cytoplasm/nucleus and mitochondria at 12 h compared to 0 h postmortem, respectively. However, 2, 6 and 10 proteins were found in the extracellular, cytoplasm/nucleus, and mitochondria at 48 h compared to 12 h postmortem, respectively. We also analyzed the functional enrichment of the differentially expressed proteins at the level of GO classification. The functional classification enrichments indicated that most differentially expressed proteins were involved in cell differentiation, regulation of apoptotic signaling pathway and oxidation-reduction process between 0 h and 12 h postmortem, and in apoptotic process, regulation of programmed cell death (apoptosis), regulation of apoptotic process and regulation of apoptotic signaling pathway between 12 h and 48 h postmortem (Figure 3c,d).

### 3.4. Differentially Expressed Protein Profiles and Protein-Protein Interaction Network

**Figure 4:**
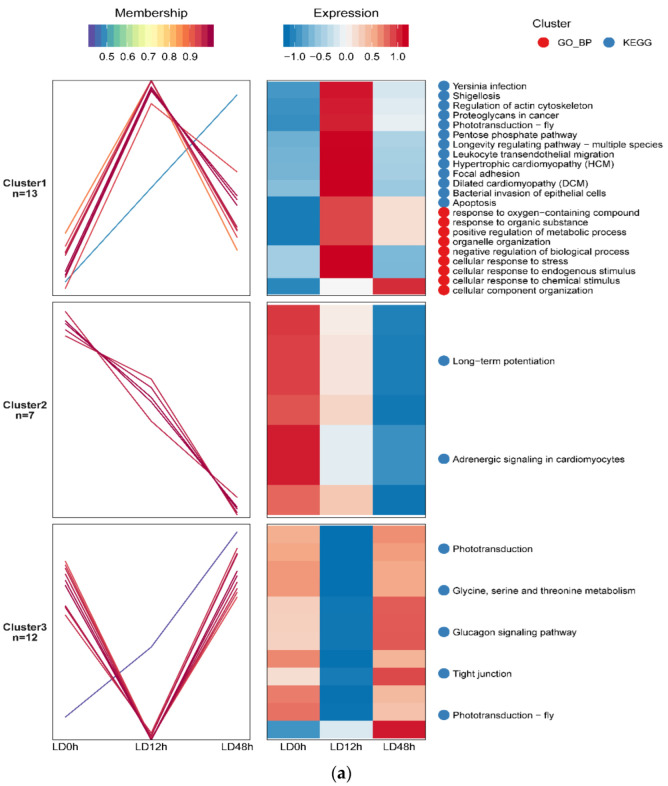

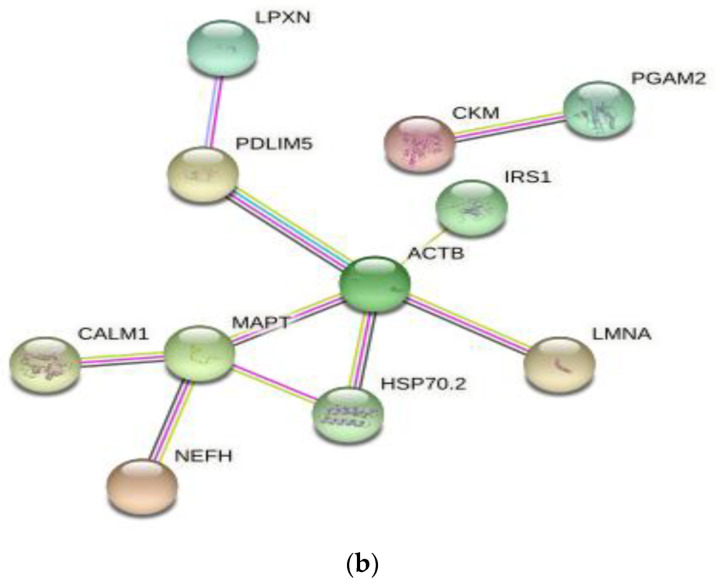
(**a**) Mfuzz analysis of differentially expressed protein profiles in postmortem pork muscle. The shade of red and blue represents high expression and low expression, respectively. (**b**) Protein–protein interaction (PPI) networks of differentially expressed proteins in postmortem pork muscle. The interaction network only presented proteins with connections.

The protein expression profiles and pathway enrichment analyses, which identified the differentially expressed proteins and signaling pathways in postmortem muscles, were performed through GO and KEGG bioinformatic resources, with special attention to the factors involved in apoptosis. A total of 32 differentially expressed proteins at the three postmortem time points (0, 12 and 48 h; Supplemental Table S2) were divided into three clusters, as shown in Figure 4a. The apoptotic process was exhibited in cluster 1, in which the protein phosphorylation level increased and then decreased or increased during postmortem aging. The STRING 11.0 database identified two cluster networks, which included 11 functional interactions of differentially expressed proteins (Figure 4b). Four interacting proteins, namely, ACTB, LMNA, MAPT and HSP70 (HSPA1B), were highly involved in apoptotic pathway. LPXN (PXN) was involved in response to oxidative stress pathway. CALM1 was involved in the calcium signaling pathway, and CKM and PGAM2 were related to the ATP metabolic process. The results showed that apoptosis- and oxidative stress-related proteins, as well as calcium signaling-related proteins, formed the most closely connected network. 

### 3.5. Differential Expression of Phosphoproteins in Postmortem Muscle

**Figure 5:**
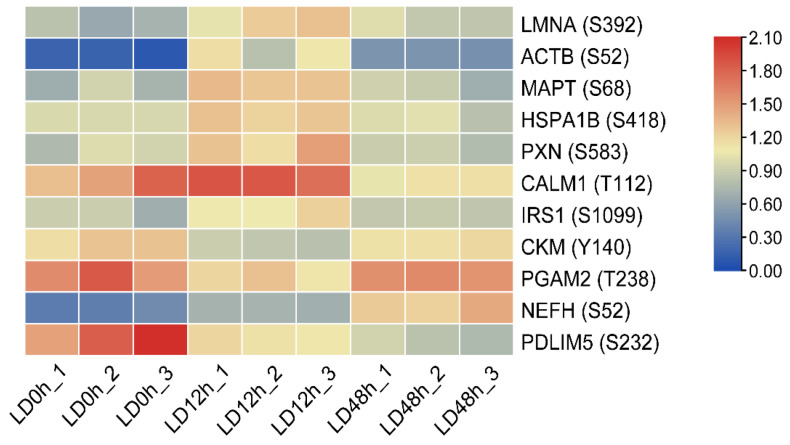
Heatmaps of interacting differentially expressed proteins in postmortem pork muscle. The shade of red and blue represents high expression and low expression, respectively.

A heat map consisting of interacting proteins (Table 1) was constructed to further investigate the levels of phosphoproteins involved in apoptosis, oxidative stress and calcium signaling pathway. From Figure 5, the levels of apoptosis-related proteins (LMNA, ACTB, MAPT and HSPA1B) were increased at 0 h to 12 h postmortem but decreased at 12 h to 48 h postmortem, which was consistent with cluster 1 of the differentially expressed proteins (Figure 4a). The phosphorylation levels of PXN and CALM1, which were involved in oxidative stress and calcium signaling pathway respectively, also increased and then decreased during postmortem aging. The results also showed that CKM and PGAM2, which were involved in the ATP metabolic process, decreased at 0–12 h but increased at 12–48 h postmortem.

## 4. Discussion

The mitochondrial apoptosis pathway involves mitochondrial dysfunction that leads to the release of apoptotic proteins, and thereby leading to apoptosis [21]. MMP has been reported to be closely associated with mitochondrial dysfunction [22]. Besides, postmortem aging entails oxidative stress that inevitably generates ROS [23]. Excessive levels of ROS likely induce MMP resulting in mitochondrial apoptosis [7]. In addition, the accumulation of ROS leads to the mitochondrial Ca^2+^ overload, which induces mitochondrial dysfunction and subsequent apoptosis [6]. Similarly, this study found that the MMP and ROS were increased at 6 h to 48 h postmortem, which were also in agreement with our previous report [7]. The mitochondrial Ca^2+^ level was increased at 6 h to 24 h postmortem, which was supported by the findings of apoptosis mediated via enhanced mitochondrial Ca^2+^ level at early aging period [18]. Further, when the apoptotic factors (such as cytochrome c) is released, it recruits ATP to activate caspases [9]. In this study, the ATP level was decreased at 0 h to 24 h postmortem, which was supported by the findings that MMP results in mitochondrial dysfunction during ATP depletion [24]. Meanwhile, ATP hydrolysis contributes to the apoptotic process in postmortem muscle [25]. Accordingly, the increased MMP, Ca^2+^ level and ROS content at 0 h to 48 h as well as decreased ATP level at 0 h to 24 h postmortem suggested mitochondrial dysfunction-mediated apoptosis at early postmortem pork muscle. 

The present study also suggested that caspases are activated in postmortem pork muscle and caspase-9 activation occurs prior to the caspase-3 activation, which coincides with our previous study in beef muscle [9]. The results were also corroborated by the findings of the activation of caspase-9 upstream of caspase-3 in mitochondrial apoptosis [1]. Caspase-3 is the executor of apoptosis and activation of caspase-3 directly initiates apoptotic events [21]. Several studies have indicated the significant roles of apoptosis in meat tenderization [6,17,18,26]. In this study, it was observed that the pork LD muscle exhibited apoptotic potential at early 0 h to 48 h postmortem, and showed more tenderness at late postmortem. The underlying mechanism of apoptosis-mediated meat tenderization needs to be further clarified.

The rapid development of novel proteomic approaches in the meat sciences has enabled the investigation of the underlying mechanisms associated with meat quality [27]. Phosphoproteomic analysis was used to elucidate the mechanism of phosphorylation regulating apoptosis-mediated meat tenderization. The present study indicated that apoptosis occurred at early 0 h to 48 h postmortem aging. Accordingly, phosphoproteomic analysis at 0 h to 48 h postmortem was conducted. A total of 82 differentially expressed proteins were mainly found in the nucleus, cytoplasm, plasma membrane and extracellular at 0 h to 12 h postmortem, whereas 144 were found in the nucleus, cytoplasm, plasma membrane and mitochondrion at 12 h to 48 h postmortem, indicating that protein phosphorylation is prone to occur in mitochondria with prolonged postmortem aging time. Mitochondria play central roles in apoptosis [26]. In the current study, ABCB8, LTBP2 and BCL2L13 were identified in mitochondria at 12 h to 48 h postmortem, and they were involved in ATP binding, calcium binding and apoptotic signaling, respectively (Supplemental Table S1). Apoptosis is a type of programmed cell death that requires ATP. Specifically, cytochrome c recruits pro-caspase-9 and activates caspase-9 and caspase-3 in the presence of ATP or dATP, thus leading to apoptosis [6]. Ca^2+^ promotes apoptotic protein release by acting on the mitochondrial membrane [28,29]. The ATP and calcium signaling pathway were also supported by the results of increasing Ca^2+^ level and decreasing ATP level in this study. In addition, BCL family proteins, which are involved in the permeabilization of the mitochondrial membrane, promote apoptosis in the postmortem muscle [4]. Thus, it was speculated that the phosphorylation of mitochondrial proteins, which regulated apoptosis, involves ATP binding, calcium binding and apoptotic signaling at 12 h to 48 h postmortem. 

It was further found that those differentially expressed proteins were highly involved in cell differentiation, oxidation-reduction processes, and the regulation of apoptotic signaling pathway at 0 h to 12 h postmortem and in the regulation of the apoptotic process, programmed cell death, and the apoptotic signaling pathway at 12 h to 48 h postmortem, indicating that the apoptosis signal pathway was mainly observed at 12 h to 48 h postmortem, which was in agreement with the results of our previous study that showed apoptotic nuclei at 12 h postmortem and an increased number of apoptotic nuclei with prolonged postmortem time [9]. Postmortem aging is a process of oxidative stress, and skeletal muscles have well-developed antioxidant enzyme systems that protect the cells from oxidative stress [30]. Consistently, the current study identified the phosphorylated proteins involved in oxidation-reduction process at 0 h to 12 h postmortem. It was also supported by the results of ROS content in this study. The decreased ROS content at 0 h to 6 h was due to the ability of the antioxidant enzyme system to eliminate part of the ROS at early aging period. Nevertheless, the homeostasis between the oxidative and anti-oxidative defense system is disturbed with the deterioration of the intracellular environment, thus resulting in ROS accumulation and further leading to apoptosis [6]. Accordingly, phosphorylation regulating apoptosis occurred at early postmortem (0–12 h) and mainly occurred at late postmortem (12–48 h).

From the PPI network of the differentially expressed proteins at the three postmortem time points, oxidative stress-related proteins and calcium signaling-related proteins were closely associated with apoptosis. Among all the critical differentially expressed proteins, we were most interested in HSP70 (HSPA1B), ACTB and MAPT. Previous studies have demonstrated that heat shock proteins (HSPs) participated in stress resistance and apoptosis, and HSPs with anti-apoptotic activity play an important role in the protection of cells and structures [31]. Stress can induce the quick phosphorylation of HSPs, and phosphorylated HSPs protect cells that are exposed to various stress factors, thus inhibiting stress-induced apoptosis [32]. In this study, the phosphorylated HSPA1B level increased at 0 h to 12 h postmortem and then decreased at 12 h to 48 h postmortem. Similarly, Huang et al. [10] reported the decreased phosphorylation levels of HSP27, HSPβ-6 and HSP90 at late postmortem. In a gel-based study, Chen et al. [14] reported that the phosphorylation levels of HSPα and HSP70 increased within 4 h but decreased at 4 h to 24 h in postmortem ovine muscle. Muscles are immediately subjected to oxidative stress after slaughter, which indicates that apoptosis occurs in postmortem muscle and mainly occurs within 48 h [17]. The high levels of phosphorylated HSPs at early postmortem may be an adaptive response to slaughter stress, thus protecting muscle cells from apoptosis caused by stress [10]. With prolonged postmortem aging, oxidative stress causes ROS accumulation and induces protein dephosphorylation, thus leading to apoptosis [18]. The global phosphorylation of HSP70 has likely contributed to the increase in its activity [33]. In fact, higher HSP expression is indicative of higher apoptotic activity [31]. Dephosphorylation of HSP70 at 12 h to 48 h induced the loss of activity of HSP70, forced apoptosis in postmortem muscle and subsequently affected meat tenderness. Li et al. [34] confirmed that phosphorylated HSPs prevented HSP degradation and promoted anti-apoptotic processes during postmortem. Furthermore, ACTB (actin) has been reported to be involved in apoptosis, as actin interacts with phosphorylated HSPs and triggers HSP activity [35].

Interestingly, ACTB and MAPT were also not involved in apoptosis, but were involved in muscle contraction from the present study. Skeletal muscle contraction is defined as continuous tension that accelerates the interdigitation of sarcomeric thin and thick filaments under the exhaustion and the release of Ca^2+^ [36]. Muscle contraction contributes to meat tenderness, and actin and microtubule are myofibrillar proteins that mediate muscle contraction [37,38]. For the proteins, decreased phosphorylation levels at 12 h to 48 h postmortem were detected for ACTB (actin) and MAPT (microtubule-associated protein). The expression of β-actin and microtubule assembly-related proteins in postmortem muscle were also studied by Chen et al. [14] and Huang et al. [10], respectively. A previous study has indicated that phosphorylation stabilizes myofibrillar proteins [39], whereas dephosphorylation degrades these proteins, thus contributing to tenderness [14,40]. The decreased phosphorylation levels of ACTB and MAPT at 12 h to 48 h postmortem was indicative of the increased degradation of these two proteins and consequently meat tenderness, which is in agreement with the decreased phosphorylation level of HSP70 at 12 h to 48 h postmortem. Equally important is that muscle contraction is an indicator of the onset of rigor mortis, which is executed by the phosphorylation of muscle contraction-related proteins [10]. Thus, the increased phosphorylation levels of ACTB and MAPT within 12 h might be in response to the contraction caused by rigor mortis.

## 5. Conclusions

In conclusion, the MMP increased at 6 h to 48 h, mitochondrial Ca^2+^ levels increased at 6–24 h but decreased at 24–48 h, mitochondrial ATP levels decreased at 0–24 h, mitochondrial ROS content decreased at 0–6 h and increased at 6–48 h, the caspase-9 activity reached a maximum level at 24 h and caspase-3 activity reached maximum at 48 h, and the shear force was increased at 0–12 h but decreased at 12–120 h postmortem, suggesting that the pork LD muscle exhibited apoptotic potential within early 48 h postmortem aging, showing more tenderness at late postmortem. The MS-based quantitative phosphoproteomic analysis of postmortem pork muscle identified 82 and 144 differentially expressed proteins at 0–12 h and 12–48 h postmortem, respectively. Most of these proteins were involved in cell differentiation, the oxidation-reduction process, and apoptotic signaling at 0–12 h postmortem and in the regulation of apoptotic process, programmed cell death, and apoptotic signaling at 12–48 h postmortem, suggesting that phosphorylation regulated apoptosis occurred at early postmortem (within 12 h) and mainly occurred at 12 h to 48 h postmortem. The subcellular localization of these proteins in mitochondria indicated that protein phosphorylation regulated apoptosis by modulating ATP and calcium bindings as well as apoptotic signaling at 12 h to 48 h postmortem. Moreover, differential expression of phosphoproteins demonstrated that phosphorylation regulated oxidative stress-induced apoptosis and rigor mortis, thereby promoting the development of meat tenderness.

## Figures and Tables

**Table 1:** Phosphorylation levels of interacting differentially expressed proteins and their involved signaling pathways in postmortem pork muscle.

Protein Accession	Posit-ion	Amino Acid	Protein Description	Gene Name	Signaling Pathway	LD 0 h	LD 12 h	LD 48 h
A0A480NRZ3	392	S	Lamin isoform A	LMNA	Regulation of apoptotic signaling pathway	0.727	1.201	0.905
A0A5S6I3N7	52	S	Actin, cytoplasmic 1	ACTB	Apoptosis	0.144	1.023	0.495
A0A287B830	68	S	Microtubule-associated protein	MAPT	Apoptotic process	0.778	1.307	0.830
Q6S4N2	418	S	Heat shock 70 kDa protein 1B	HSPA1B	Regulation of apoptotic signaling pathway	0.970	1.271	0.942
A0A5G2QL63	583	S	Paxillin	PXN	response to reactive oxygen species	0.898	1.316	0.860
A0A5G2QTD3	112	T	Calmodulin-1 isoform X1 (Fragment)(predicted)	CALM1	Calcium signaling pathway	1.532	1.835	1.113
I3LLF1	1099	S	Insulin receptor substrate 1	IRS1	Aging	0.827	1.132	0.863
Q5XLD3	140	Y	Creatine kinase M-type	CKM	ATP metabolic process	1.250	0.855	1.158
B5KJG2	238	T	Phosphoglycerate mutase	PGAM2	ATP metabolic process	1.654	1.208	1.575
F1RFH3	52	S	Neurofilament heavy polypeptide	NEFH	Biological regulation	0.379	0.706	1.306
A0A287A7G2	232	S	PDZ and LIM domain 5	PDLIM5	Cell differentiation	1.802	1.148	0.839

## Data Availability

Data is contained within the article.

## Supplementary Materials

The following supporting information can be downloaded at: https://www.mdpi.com/article/10.3390/foods11233751/s1, Table S1: Phosphorylated differentially expressed proteins in postmortem muscle at 0 h to 48 h; Table S2: Differentially expressed proteins at the three postmortem time points (0 h, 12 h, and 48 h).
